# Estimation of the basic reproduction number (R0) for the novel coronavirus disease in Sri Lanka

**DOI:** 10.1186/s12985-020-01411-0

**Published:** 2020-10-07

**Authors:** Samath Dharmaratne, Supun Sudaraka, Ishanya Abeyagunawardena, Kasun Manchanayake, Mahen Kothalawala, Wasantha Gunathunga

**Affiliations:** 1 Faculty of Medicine, University of Peradeniya, Kandy, Sri Lanka; 2 Department of Health Metrics Sciences, School of Medicine, Institute for Health Metrics and Evaluation, University of Washington, WA Seattle, USA; 3 Faculty of Engineering, University of Ruhuna, Matara, Sri Lanka; 4 National Hospital, Kandy, Sri Lanka; 5 Department of Community Medicine, Faculty of Medicine, University of Colombo, Colombo, Sri Lanka

**Keywords:** Basic reproduction number, R0, Coronavirus

## Abstract

**Background:**

The basic reproduction number (R0) is the number of cases directly caused by an infected individual throughout his infectious period. R0 is used to determine the ability of a disease to spread within a given population. The reproduction number (R) represents the transmissibility of a disease.

**Objectives:**

We aimed to calculate the R0 of Coronavirus disease-2019 (COVID-19) in Sri Lanka and to describe the variation of R, with its implications to the prevention and control of the disease.

**Methods:**

Data was obtained from daily situation reports of the Epidemiology Unit, Sri Lanka and a compartmental model was used to calculate the R0 using estimated model parameters. This value was corroborated by using two more methods, the exponential growth rate method and maximum likelihood method to obtain a better estimate for R0. The variation of R was illustrated using a Bayesian statistical inference-based method.

**Results:**

The R0 calculated by the first model was 1.02 [confidence interval (CI) of 0.75–1.29] with a root mean squared error of 7.72. The exponential growth rate method and the maximum likelihood estimation method yielded an R0 of 0.93 (CI of 0.77–1.10) and a R0 of 1.23 (CI of 0.94–1.57) respectively. The variation of R ranged from 0.69 to 2.20.

**Conclusion:**

The estimated R0 for COVID-19 in Sri Lanka, calculated by three different methods, falls between 0.93 and 1.23, and the transmissibility R has reduced, indicating that measures implemented have achieved a good control of disease.

## Introduction

Sri Lanka reported its first patient with the novel Coronavirus disease-2019 (COVID-19) on the January 27th, 2020, a Chinese female visiting the country [1]. The first local patient was reported on March 11th, 2020 thus bringing about unprecedented changes in the daily life in the country [2]. Rigorous measures were implemented to reduce the spread of the disease, however despite these measures new patients were reported almost every day [3–7]. The rise in the daily total number of patients did not however show a marked exponential rise but remained steady after the first week [3]. This pattern lasted up to April 19th, 2020 following which, the detection of two clusters of patients led to a sudden increase in the number of cases [8, 9]. However, since then due to government interventions including meticulous testing, the numbers have once again started to reduce [3]. In this paper, we aimed to calculate the R0 for the spread of COVID-19 in Sri Lanka and to describe the variation of R with its implications to the prevention and control of the disease.

The Basic Reproduction Number or R0 is defined as the average number of secondary infections which can be caused by a patient, in a completely susceptible population, throughout his infectious period [10]. Therefore, R0 is a dimensionless number and an indicator of the contagiousness of an pathogen [2]. Environmental conditions and the pattern of human interactions affect the transmission of disease and thereby affect the R0. Hence, the R0 is not a constant for a pathogen itself, but rather a constant for a pathogen in each population [11].

The two uses of the R0 are, to assess the ability of an infectious disease to invade the community (when the R0 of a disease is greater than 1, the infection will spread, as it indicates that one infected individual will spread the disease to more than one individual) and to determine the fraction of the community which should be vaccinated in order to prevent the growth of the epidemic [10].

The R0 should not be confused with the reproduction number (R), which is the average number of secondary cases of disease caused by a single infected individual over the infectious period. Unlike R0, R varies with time and is commonly used to describe the transmissibility of the pathogen during an epidemic. The variation of R over time reflects effectiveness of control measures and highlights when the control efforts need to be intensified. A value of R below one, close to zero reflects the success of the control measures in controlling the epidemic [12]. We aimed to calculate R0 for COVID-19 in Sri Lanka and to describe the variation of R since the report of the first local case.

## Materials and methods

The total number of confirmed COVID-19 patients who tested positive with the reverse transcriptase polymerase chain reaction, reported daily, was extracted from the daily situation reports of the Epidemiology Unit, Ministry of Health, Sri Lanka [3]. The mean and the standard deviation of the serial interval of COVID-19 (the time between the onset of symptoms in a primary case and the onset of symptoms in secondary cases) was taken as 3.96 days and 4.75 days respectively [13].

As there were new clusters of patients identified after April 19th, 2020, which changed the pattern of spread, two R0s were calculated, for the data up to April 19th, 2020 and secondly for data up to April 30th, 2020, from the day of the first reported local patient.

We utilized a compartmental model with 3 compartments as ‘Susceptible’, ‘Infected’ and ‘Removed (recovered and dead)’ (SIR). The model divides the population into three compartments and evaluates the dynamics of each compartment using a mathematical model [10].

Therefore, if the population size is N, at any given time, N = S + I + R = constant. It is assumed that the transmission and removal rates are constant and that there are no demographical changes within the population. Individuals in the ‘S’ compartment can progress to the ‘I’ compartment and individuals from the ‘I’ compartment can progress to ‘R’. The way this progression takes place can be explained by following differential equations [10].

$$\begin{aligned} \frac{dS}{{dt}} & = - \frac{\beta SI}{N} \\ \frac{dI}{{dt}} & = \frac{\beta SI}{N} - \gamma I \\ \frac{dR}{{dt}} & = \gamma I \\ \end{aligned}$$

The terms are defined as follows.

S: The number of individuals in the susceptible group at a given time.
I: The number of individuals in the infected group at a given time.
R: The number of individuals in the removed group at a given time.

The terms, *dS/dt*, *dI/dt* and *dR/dt* denote the change in the ‘S’, ‘I’ and ‘R’ compartments with time.

N: Population size.
Β: Effective contact rate (The number of cases caused by one infected individual, effectively, in a unit time).
ƴ: Rate of removal.

In a completely susceptible population, the number of new infections produced by the index case is equal to the effective contact rate times the average infectious period $\left( {\beta \times \frac{1}{\gamma }} \right),$ which, by definition is R0 [10].

The parameters β, ƴ and N for the two SIR models, which gave the least root mean squared error (RMSE) for the total cases reported daily by the Epidemiology Unit, were estimated using MATLAB, a multi-paradigm numerical computing environment and proprietary programming language [14]. In this manner, R0 was calculated for both sets of data, using the model parameters, and the RMSE was used to determine the best representative model, and its R0, out of the two values.

This value of R0 was further corroborated by the exponential growth method and the maximum likelihood estimation method, using the ‘R0, Estimation of R0 and Real-Time Reproduction Number from Epidemics’ package, for R language in statistical computing [15].

The reproduction number R, can be estimated by the ratio of the number of new infections generated at time step *t*, to the total infectiousness of infected individuals at time *t* [16]. The variation of R in Sri Lanka over time was calculated using a package ‘EpiEstim’ created by Cori et al., for R language in statistical computing. A sliding time window of 7 days was used to minimize the variation of R and to obtain a narrow 95% credible interval, assuming the reproduction number is constant within that time window. This method is based on a Bayesian statistical inference assuming a gamma prior distribution for R [12].

## Results

**Fig. 1:**
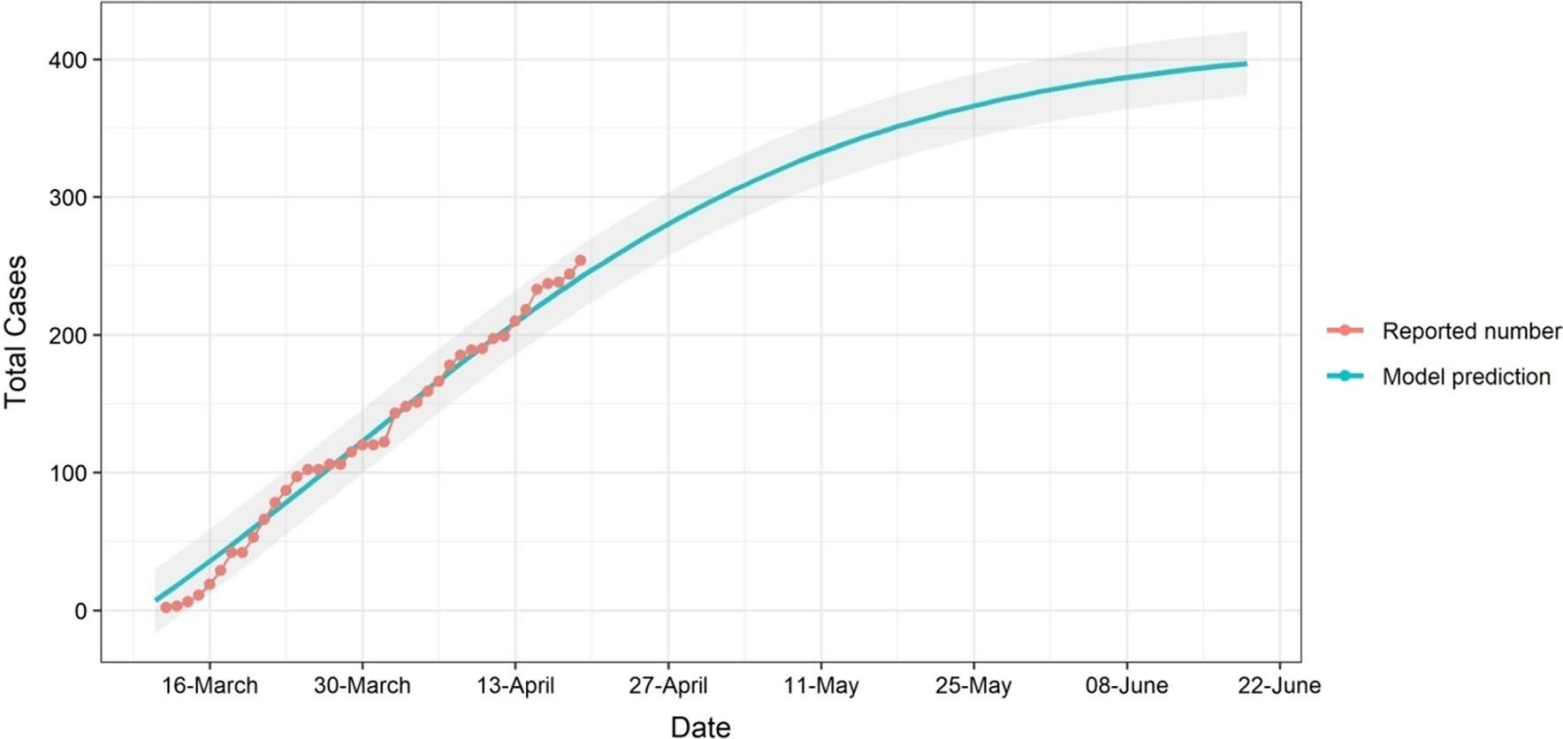
The reported number of total cases (for data up to April 19th, 2020) and the predicted number of total number of cases by the model (RMSE = 7.72124)

The R0 obtained for the data up to the April 19th, 2020, using estimated SIR model parameters was 1.02 [confidence interval (CI) of 0.75–1.29] with an RMSE of 7.72. The model prediction for total number of cases and the total number of cases actually reported are illustrated in Fig. 1.

**Fig. 2:**
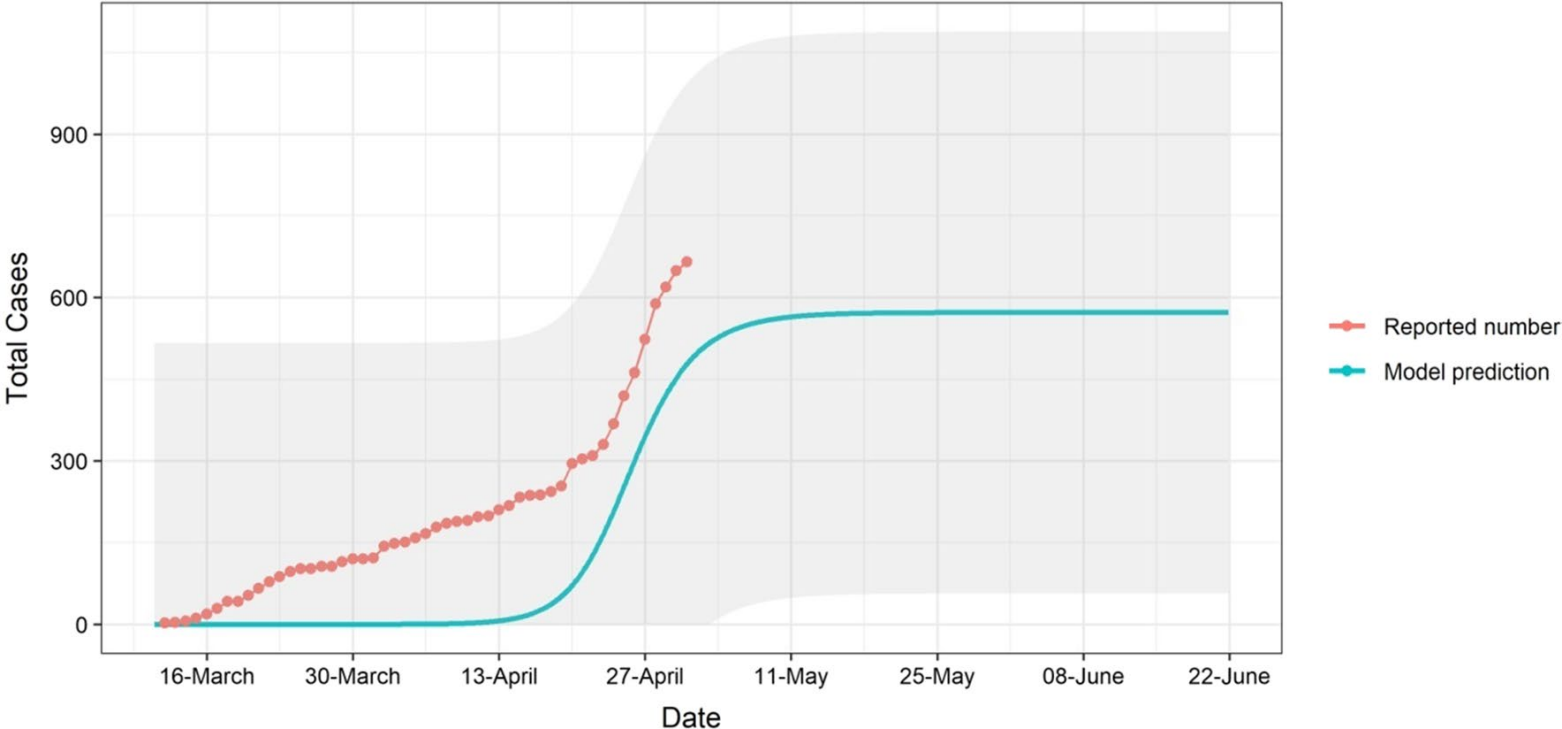
The reported number of total cases (for data up to April 30th, 2020) and the predicted number of total number of cases by the model (RMSE = 172.444)

The R0 calculated by the model using data up to April 30th (RMSE of 172.44) was 1.66 (CI of 0.98–2.33). Figure 2 illustrates the prediction for total number of cases by the model in comparison to the total number of cases reported.

**Fig. 3:**
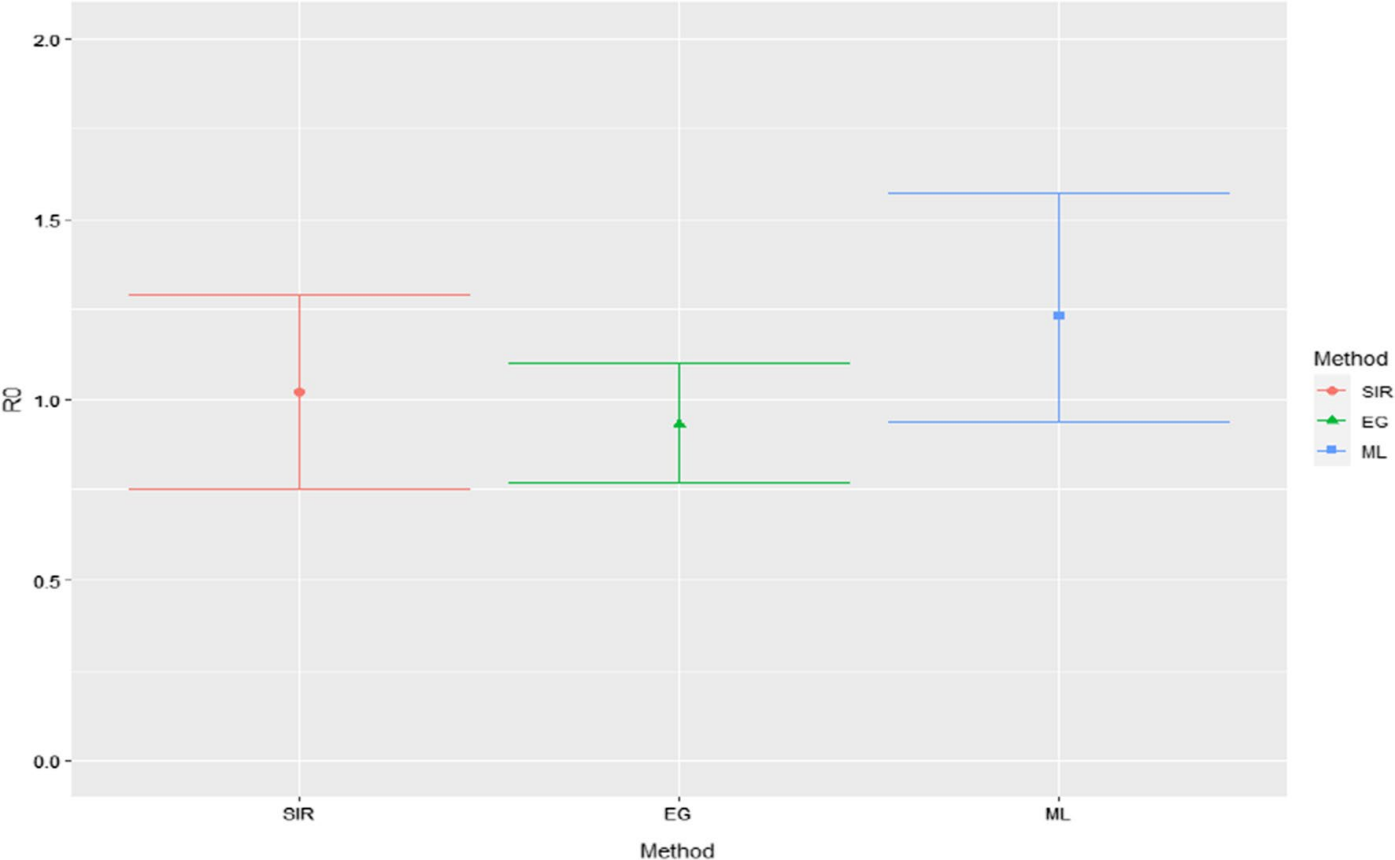
R0 estimates and 95% confidence intervals from the SIR model, exponential growth method (EG) and the maximum likelihood (ML) estimation method

The low RMSE of the first model indicates that the model is more representative of the spread in Sri Lanka. The exponential growth rate method and the maximum likelihood estimation method yielded an R0 of 0.93 [confidence interval (CI) of 0.77–1.10] and an R0 of 1.23 (CI of 0.94–1.57) respectively, when applied to this data set. This is illustrated in Fig. 3.

**Fig. 4:**
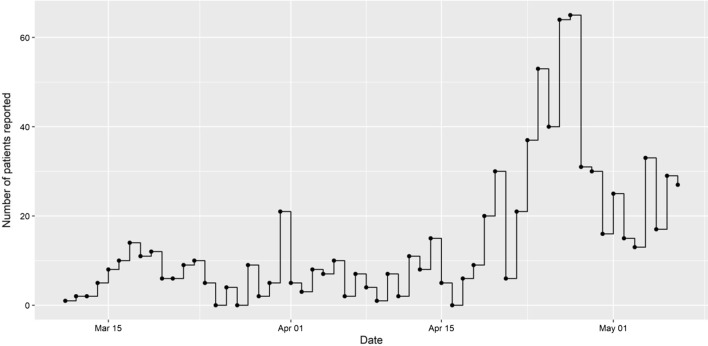
The total number of cases reported daily from March 11th, 2020, to May 7th, 2020

**Fig. 5:**
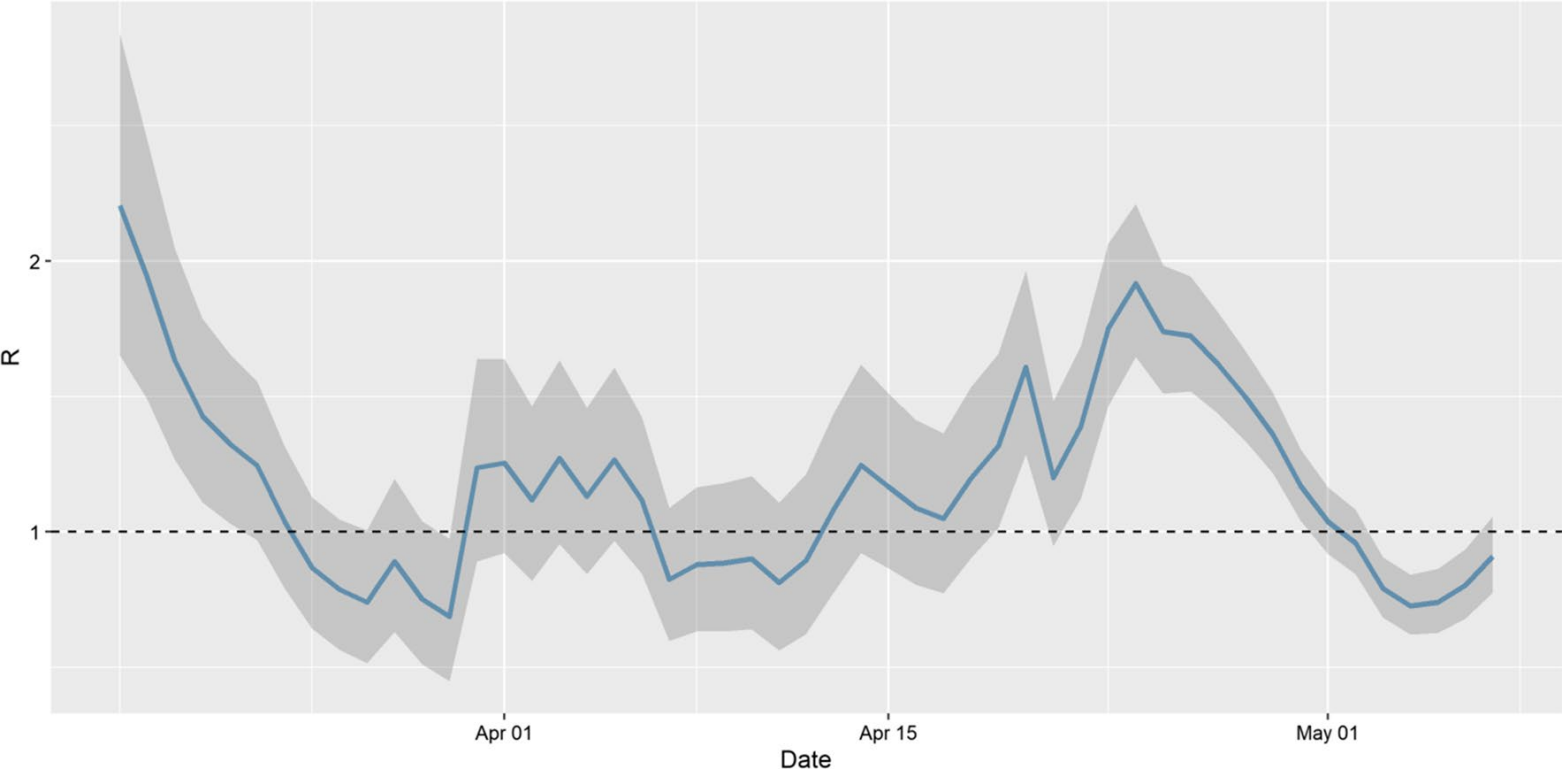
The variation of R, the transmissibility of COVID-19, from the March 11th, 2020 to the May 7th, 2020

The real time reproduction number (R) was found to show considerable variation with time, ranging from 0.69 (95% credible intervals of 0.45–0.97) to 2.20 (95% credible intervals of 1.65–2.83). It is evident that implementation of initial control measures reduced the transmissibility, which rose once again with the detection of the two clusters of cases. However, despite this setback, the transmissibility R is reducing once again. The daily total number of patients is illustrated in Figs. 4 and 5 depicts the variability of R with time.

## Discussion

The first model, with the data up to April 19th, 2020 estimated the R0 as 1.02 whereas the second model using data up to the April 30th, 2020 estimated the R0 as 1.66, using the model parameters. The first model’s estimate of an R0 of 1.02 is more representative, with an RMSE of 7.72 for the cumulative number of patients. The second model utilizing the SIR model calculated a higher RMSE indicating that the observed cumulative number of patients does not fit well for the model predictions. The inaccuracy of this second model is likely to be due to the detection of two clusters of patients, one in a residential neighborhood and the other in the Navy reported from the April 20th, 2020 to date.

Currently however, rigorous measures that have been put in place were able to limit the spread of the disease from these two clusters and the numbers reported each day have once again begun to decline [3]. This is also evidenced by the gradual reduction in R. Hence, it follows that the increased number of patients reported within these 10 days can be taken as a cluster epidemic, not in line with the spread seen in Sri Lanka in the previous 42 days following the diagnosis of the first local patient on March 11th, 2020 [2].

**Fig. 6:**
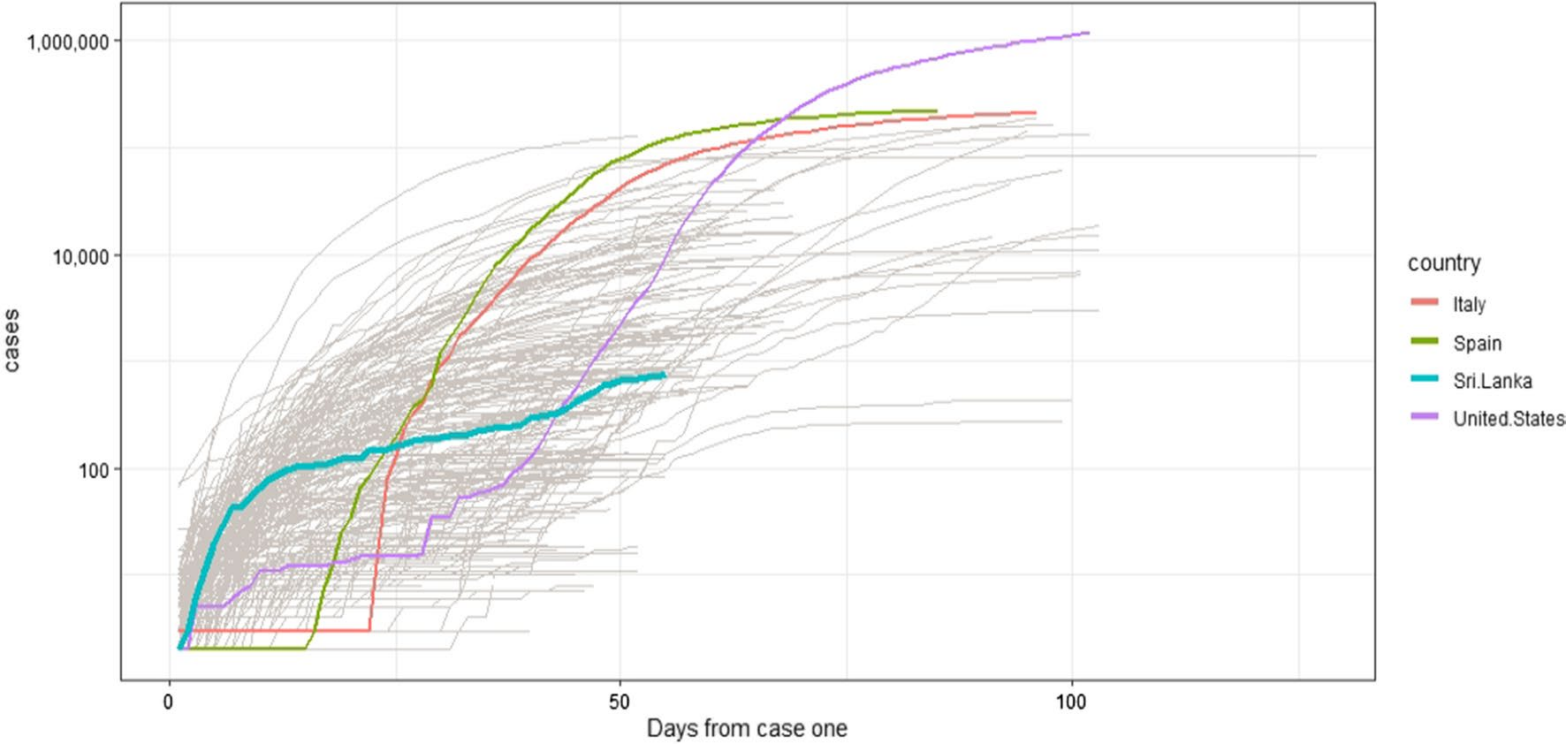
The number of confirmed cases of COVID-19 reported from the first day of the first case for Italy, Spain, USA and Sri Lanka, using a log scale

The utilization of all three methods estimates the R0 to be between 0.93 and 1.23. China reported a R0 of 2.2, [17] and Italy as 2.4–3.1 [18]. The Fig. 6 represents the daily total cases reported in countries worldwide, since the day of their first reported case (in log scale). The United States of America (USA), Italy, Spain and Sri Lanka are highlighted [19]. The impact of the preventive measures implemented in Sri Lanka is evident.

The collaborative efforts of the tri-forces, police, intelligence services and healthcare workers under the guidance of the President of Sri Lanka, from the beginning of the epidemic, were instrumental in preventing the spread of the disease in Sri Lanka. Implementation of nationwide curfews, restriction of flights, the quarantine of those returning from abroad along with meticulous contact tracing by the intelligence services, home isolation protocols are merely the tip of the iceberg in these efforts taken to prevent the spread of the disease [6–9]. The implementation of social distancing protocols and curfews also contributed significantly in reducing the contact rate of an infected individual. A never ending circle of test-catch-quarantine followed by contact trace-test-catch-quarantine were the cornerstones of the measures initiated and implemented in Sri Lanka, giving rise to suppression of spread and containment of the disease [20].

The limitations of this study are as follows. R0 was calculated using the SIR model which assumes that the members of the population mix homogeneously and transmission and removal rates are constant. Since several interventions were implemented during the epidemic, to limit the spread of the disease, both population mixing and transmission rates may have not been constant. The exponential growth method and the maximum likelihood estimation should be calculated on the period of exponential growth. The exponential growth phase was not as marked in Sri Lanka when compared with some other countries worldwide, however, the calculation was carried out on the exponential phase seen in Sri Lanka, which was selected following a sensitivity analysis for an exponential regression model for the cumulative number of daily reported cases.

## Conclusion

In conclusion, our estimated R0 for COVID-19 in Sri Lanka falls between 0.93 and 1.23. This along with the reduction of the transmissibility, R, reflects a relatively good control of disease spread. This indicates that a preventive strategy, based on the collaboration of the military, intelligence services, healthcare workers and the police is effective even for developing countries, to combat a pandemic.

## Data Availability

Data used for this study is available at the Epidemiology Unit, Ministry of Health, Sri Lanka.

## Abbreviations

R0: Basic reproduction number
R: Reproduction number
COVID-19: Coronavirus disease-2019
RMSE: Root mean squared error
CI: Confidence interval
SIR: Susceptible, infected and removed
USA: United States of America
